# Plasma membrane integrity: implications for health and disease

**DOI:** 10.1186/s12915-021-00972-y

**Published:** 2021-04-13

**Authors:** Dustin A. Ammendolia, William M. Bement, John H. Brumell

**Affiliations:** 1 Cell Biology Program, Hospital for Sick Children, 686 Bay Street PGCRL, Toronto, ON M5G 0A4 Canada; 2 Department of Molecular Genetics, University of Toronto, Toronto, ON M5S 1A1 Canada; 3 Center for Quantitative Cell Imaging and Department of Integrative Biology, University of Wisconsin-Madison, Madison, WI 53706 USA; 4 Institute of Medical Science, University of Toronto, Toronto, ON M5S 1A1 Canada; 5 SickKids IBD Centre, Hospital for Sick Children, Toronto, ON M5G 0A4 Canada

**Keywords:** Plasma membrane, Membrane damage, Lipid peroxidation, Pore formation, Membrane repair, Vesicle trafficking, Cell biology, Tissue injury, Disease

## Abstract

Plasma membrane integrity is essential for cellular homeostasis. In vivo, cells experience plasma membrane damage from a multitude of stressors in the extra- and intra-cellular environment. To avoid lethal consequences, cells are equipped with repair pathways to restore membrane integrity. Here, we assess plasma membrane damage and repair from a whole-body perspective. We highlight the role of tissue-specific stressors in health and disease and examine membrane repair pathways across diverse cell types. Furthermore, we outline the impact of genetic and environmental factors on plasma membrane integrity and how these contribute to disease pathogenesis in different tissues.

## Plasma membrane integrity

Confinement of a cell from its surrounding environment is a universal trait of microscopic life. The plasma membrane fulfills this role whereby its integrity is vital for cell function and survival. Accordingly, plasma membrane architecture and composition varies to provide resistance to injury in different cellular contexts. Despite this protection, various factors (herein referred to as stressors) present in the extra- and intra-cellular environment can induce chemical disruptions or physical breaches in the plasma membrane. Although not all wounds result in cell death, even sublytic damage can vastly change the intracellular landscape through cytosolic leakage and exposure to the outside environment.

In vivo, plasma membrane damage is encountered during normal physiological events such as muscle contraction and locomotion [1–3]. In these situations, sublytic damage can prove beneficial by stimulating paracrine signaling to shape the tissue environment. Alternatively, plasma membrane damage inflicted by microbial pathogens and immune cells can have deleterious consequences on cell fate during infection and inflammation [4, 5]. The prevalence of membrane damage across functionally distinct processes, from cell death to cancer cell migration, exemplifies how plasma membrane integrity is a fundamental aspect of cell biology [6, 7].

To deal with damage, cells are equipped with plasma membrane repair mechanisms. Ion imbalances “sound the alarm”, leading to cytoskeleton remodeling, repair factor recruitment, and vesicle trafficking, which cooperatively facilitate wound closure [8]. While several different repair pathways have been identified, our understanding of how these mechanisms cooperate to facilitate resealing remains unclear. Moreover, nearly all of the known repair mechanisms were identified based on studies of extracellular stressors, yet recent evidence highlights the importance of repair in response to internal stressors such as necroptotic pores and even endolysosomal damage [9, 10].

Plasma membrane integrity reflects resistance and repair capacity, both of which are influenced by host genetics and environmental factors. Imbalances in either of these determinants of membrane integrity can lead to disease pathogenesis. In the case of muscular dystrophies, genetic factors can dampen membrane resistance and repair whereas in other instances, such as traumatic brain injury, physical trauma can exceed a cell’s repair capacity and result in membrane lesions [11, 12]. Emerging evidence suggests that defective repair may even contribute to the pathogenesis of multifactorial diseases such as inflammatory bowel disease (IBD) [13]. Considering the plethora of factors that can dampen plasma membrane integrity, barrier maintenance should be regarded as an active cellular process that ultimately sustains tissue structure and function. While tissue damage is a common hallmark of disease, the role of plasma membrane integrity in disease initiation and progression remains understudied.

Plasma membrane damage and repair are often generalized across literature and experimental settings, which, despite advancing our mechanistic understanding of these processes, has made it difficult to infer the physiological relevance of these findings. Here, we summarize our current understanding of plasma membrane damage and repair from a whole-body perspective, including tissue-specific stressors and the influence of genetic and environmental factors on cell type-specific repair. We highlight that defects in plasma membrane resistance and repair exacerbate injury and contribute to a broad range of human diseases. Understanding wound resolution on a single-cell basis can lead to the identification of promising therapeutic targets to promote tissue regeneration in several pathophysiological states.

## Types of plasma membrane damage

Plasma membrane damage is conventionally assessed by indirect means such as the entry of cell-impermeable molecules (e.g., dextran), calcium influx, or the detection of intracellular contents in the extracellular environment [14]. Direct measures of visualizing and characterizing plasma membrane wounds are challenged by technical constraints and the rapid speed of the repair process. Nonetheless, studies using such approaches have demonstrated that plasma membrane integrity can be compromised from two distinct types of damage: chemical disruptions and physical breaches.

### Chemical disruptions

**Fig. 1:**
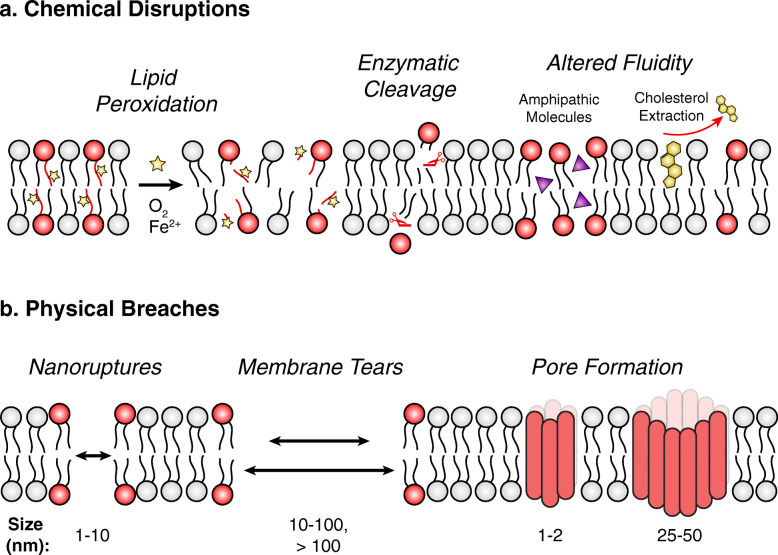
Plasma membrane damage is comprised of chemical disruptions and physical breaches. **a** Chemical disruptions of the plasma membrane can alter its biophysical properties and lead to a breach. Oxidative stress and intracellular iron promote lipid peroxidation of poly-unsaturated fatty acids leading to the removal of damaged fragments and destabilization of the plasma membrane. Membrane lipids are subject to enzymatic damage by host or foreign phospholipases. Alterations in membrane fluidity through interactions with amphipathic molecules or cholesterol extraction can weaken membrane resistance upon subsequent insult. **b** Depending on size and frequency, physical breaches elicit lytic or non-lytic damage and require active repair to restore membrane integrity. Nanoruptures result in ion imbalances and the leakage of small molecules, whereas membrane tears result in extensive leakage of cytosolic cargo. Larger membrane tears (> 100 nm) are broadly distinguished based on different repair requirements. Upon recognition of unique host receptors, pore-forming proteins can assemble into transmembrane pores that differ in terms of size, structure, and ion flux

The biochemical nature of the plasma membrane renders it susceptible to several forms of chemical disruptions (Fig. 1a). In the presence of reactive oxygen species (ROS), the oxidation of polyunsaturated fatty acids (i.e., lipid peroxidation) can lead to the release of damaged lipid fragments and eventual loss of plasma membrane integrity [15]. Lipid peroxidation is accelerated by intracellular iron via the Fenton reaction [16] whereas membrane damage is countered by cholesterol, antioxidants, and glutathione peroxidase 4 (GPX4), a central lipid repair factor [15, 17, 18]. This form of membrane injury is often detected by end products [19]; however, these measures make it difficult to infer the extent of damage on a single cell level (i.e., transient or lytic) and the influence of redox status on cell fate. Alternatively, the plasma membrane is subject to enzymatic damage (e.g., phospholipases) that can alter membrane fluidity and render cells more prone to osmotic lysis—similar to what is observed upon cholesterol extraction [20–22]. Amphiphilic molecules (e.g., drugs, alcohol) can also disrupt membrane fluidity through direct interactions whereas at lower magnitudes they potentiate oxidative stress-induced damage [23, 24].

### Physical breaches

Physical breaches of varying size and nature can compromise plasma membrane integrity (Fig. 1b). Tiny punctures (< 1 nm) do not result in a permanent breach as it is energetically favorable for lipids surrounding the lesion to undergo spontaneous resealing [25]. However, larger injuries such as nanoruptures, tears, and pores are not self-limiting and result in a physical breach. Nanoruptures are characterized as small lesions (~ 1–10 nm wide) with exposed lipids around the wound edge that arise from mechanical force and sustained chemical disruptions [26, 27]. Larger membrane ruptures, herein referred to as tears, can be broadly categorized based on their size (i.e., greater or less than 100 nm) given differences in repair requirements [28]. Membrane tears are often encountered in muscle cells following exercise or can arise from physical trauma [12, 29]. Little is known about the topology of nanoruptures and tears, and whether some regions of plasma membrane are more prone to tearing than others (e.g., influence of local lipid composition or plasma membrane-organelle contact sites).

Pore formation is the most well-characterized type of physical breach given the prevalence of pore-forming proteins in immunity and infectious disease [4, 5]. As reviewed in great detail [30], pore formation entails the recognition of protein monomers with unique host surface receptors, which triggers oligomerization and the insertion of membrane pores that vary in terms of size and ion selectivity. In comparison to larger pores (~ 25–50 nm), small pores (~ 1–2 nm) generally persist longer on the plasma membrane which has been suggested to reflect lower extents of calcium influx, and consequently, delayed calcium-dependent repair mechanisms [31–33]. Contrary to tears, pores lack membrane edges which may explain why these wounds have different repair requirements [34]. Plasma membrane composition is a major determinant of cellular resistance against pore-induced damage. For instance, the availability of surface cholesterol and its distribution in microdomains can greatly influence the extent of membrane damage by cholesterol-dependent cytolysins [35, 36].

## Extracellular and intracellular sources of plasma membrane damage

**Fig. 2:**
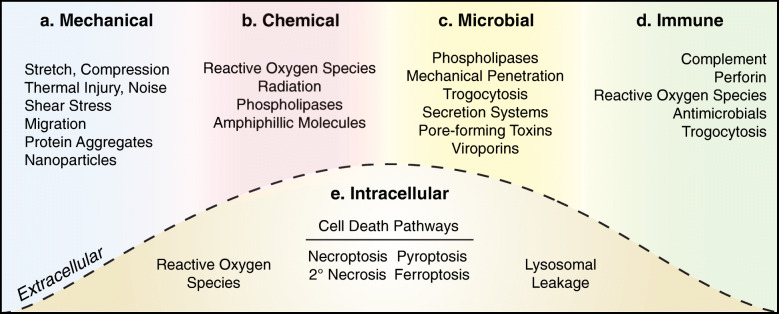
Five major sources of plasma membrane damage. These sources of damage can be highly overlapping as one type of membrane injury can lead to another. (a) Cells experience mechanical stress from physiological events (e.g., locomotion), cell migration, and through interactions with inert substances in the local environment, all of which elicit membrane damage in the form of nanoruptures and tears. (b) Reactive oxygen species present in the extracellular environment or those generated from irradiation can promote lipid peroxidation. Additional sources of chemical disruptions include amphipathic molecules (e.g., NSAIDs and alcohol), which can compromise membrane integrity either through direct interactions or indirectly via oxidative stress. (c) Microbial species employ several strategies to induce plasma membrane damage. Virulence factors can inflict chemical disruptions (e.g., phospholipases) and physical breaches (e.g., pore-forming toxins); meanwhile, larger species can also exert brute force to damage host plasma membrane. (d) Immune cells elicit membrane damage, namely through pore-forming proteins and antimicrobials, under several unique contexts such as immune surveillance and neutrophil extracellular traps. (e) Intracellular sources of plasma membrane damage include oxidative stress, which can entice lipid peroxidation, and the leakage of cytotoxic enzymes from lysosomes. In the context of cell death, many pathways employ pore-induced damage (e.g., necroptosis, secondary necrosis, pyroptosis) whereas others are characterized by chemical disruptions (e.g., ferroptosis)

Within a tissue environment, a multitude of stressors can induce plasma membrane damage through chemical disruptions and physical breaches. Here, we categorize 5 major sources of plasma membrane damage: mechanical, chemical, microbial, immune, and intracellular stressors (Fig. 2). Notably, these sources of damage are not mutually exclusive, and in many cases, one form of injury predisposes the plasma membrane to subsequent insult (e.g., chemical damage exacerbates mechanical insult) [37].

### Mechanical

In vivo, physiological events such as locomotion can generate sufficient mechanical force to elicit plasma membrane damage in muscle, bone, and skin [2, 26, 29]. Alternatively, mechanical lesions can arise from physical trauma, thermal injury, and even exposure to penetrating noise and ultrasound [12, 38–40]. In all these contexts, our characterization of membrane damage is limited to the uptake of cell-impermeable dyes; however, one can speculate as to the general mode of disruption (e.g., stretch- or compression-induced tears). We also have a very limited understanding of the frequency and size of wounds caused by physiological force, although in the case of muscle contractions, damage is speculated to be a gradient of nanoruptures and tears on the scale of micrometers [1, 11, 29].

On a cellular level, physical breaches can arise from shear stress, such as during the transit of red blood cells and platelets in circulation [41–43]. Intriguingly, malignant cancer cells harness the abundance of platelets to bolster their resistance to such shear stress [44, 45]. Upon exiting circulation, cancer cells continue to face mechanical damage during cell migration which they combat through enhanced repair efforts [7, 46–48]. Moreover, it is apparent that cells bordering sites of tissue trauma also experience strain-induced plasma membrane damage [49] which seems likely to influence the rate of tissue repair.

Mechanical damage can also arise following the interaction of the plasma membrane with inert substances (e.g., protein aggregates, inhaled nanoparticles). For instance, intrinsically disordered proteins (e.g., β-amyloid) have the propensity to aggregate in the extra- and intra-cellular environment and form pore-like structures in the plasma membrane [50]. Similar modes of mechanical disruption contribute to the nanotoxicity of silica nanoparticles and multi-walled carbon nanotubes, albeit through different mechanisms [51, 52]. Understanding these forms of nanotoxicity is of critical importance in the development of safe medical solutions based on nanotechnology [53].

### Chemical

Lipid peroxidation is a prominent stressor across tissue environments although the source of ROS varies. During hypoxia or ischemia/reperfusion (I/R) injury, inadequate blood supply leads to mitochondrial dysfunction and the generation of ROS which is further exacerbated upon reoxygenation [54]. Metabolically active tissues, such as kidney and liver, are particularly sensitive to this form of oxidative injury [55, 56]. Alternatively, lipid peroxidation can occur following irradiation [57, 58], exposure to extracellular ROS (e.g., cancer therapy) [59], and through interactions between intrinsically disordered proteins with the inner- and outer-leaflet of the plasma membrane [60, 61]. Notably, there appears to be a positive feedback cycle whereby oxidized membrane can foster protein aggregation at the cell surface to further exacerbate injury [61].

Early gastrointestinal studies revealed the ability of amphiphilic molecules such as bile acids, alcohol, and non-steroidal anti-inflammatory drugs (NSAIDs), to directly compromise plasma membrane integrity at high concentrations [62–64]. However at lower concentrations, these stressors promote oxidative stress through mitochondrial dysfunction [23, 65] and alter membrane lipid organization [66, 67]. The propensity for these stressors to induce damage is also influenced by nearby chemical species. For instance, while certain bile acids can alter membrane fluidity without an apparent breach [68], the simultaneous presence of NSAIDs can promote damaging extents of bile acid accumulation [69].

### Microbial

Microbial species have several motives to induce plasma membrane damage such as entry into the host’s nutrient-rich intracellular environment, facilitating cellular escape and spread, or eliciting host damage [70, 71]. To breach the plasma membrane, chemical disruptions can be achieved by virulence factors such as secreted sphingomyelinases and phospholipases [72]. However, the use of mechanical stress to induce a breach is far more apparent across bacteria, fungi, protozoa, and viruses.

To facilitate invasion, larger species (e.g., fungi, protozoa) exert brute force onto the host plasma membrane. During the filamentous growth of yeast, hyphae protrusions damage the plasma membrane of epithelial cells [73, 74]. Similarly, *Plasmodium* sporozoites physically penetrate through the skin epithelium causing irreversible host cell damage [75]. The onset of necrotic death in both these instances argues in favor of large plasma membrane tears. However, such crude destruction is not always the case as parasites can nibble pieces of host plasma membrane through a process called “trogocytosis” [76]. Miraculously, the host can maintain plasma membrane integrity until the point of cell death, suggesting a role for active repair pathways during this process.

As cell death is not always the desired outcome, microbes are equipped with specialized machinery to induce smaller breaches. Bacteria utilize needle-like structures (termed secretion systems) to facilitate cargo delivery into host cells through a transmembrane pore (~ 1.2–5 nm in the case of type 3 secretion systems) [71, 77, 78]. Similarly, *Microsporidia* breach host membrane using a much larger “polar tube” (~ 0.1–0.15 μm diameter) although the insertion mechanism or type of wound this inflicts is unknown [79]. Viruses also exploit plasma membrane damage: for example, adenovirus relies on a single surface protein to induce damage and facilitate entry into the host cell [80].

Mechanical damage is also achieved by pore-forming toxins; the largest class of bacterial toxins and similar members are expressed by fungi and protozoan species [81–84]. While many of these toxins are capable of inducing cell lysis, sublytic damage provides initiating cues (e.g., ion imbalances) to alter the intracellular environment and, in some cases, initiate cell death pathways [4, 31, 85]. Although distinct from bacterial pore-forming toxins, viruses similarly compromise host membrane integrity through transmembrane channels termed viroporins [86, 87].

### Immune

Plasma membrane damage is fundamental to several immune functions. Circulating complement factors can achieve cell lysis upon formation of a heteromeric pore (~ 12 nm wide) known as the membrane attack complex [88]. Under normal conditions, regulatory mechanisms protect host cells from complement-induced damage; however, autoantibodies or an overwhelming inflammatory response can promote this form of injury [89, 90]. Meanwhile, cytotoxic lymphocytes inflict sublytic damage at immune synapses through perforin pores (~ 13–20 nm wide) to enable the intracellular delivery of pro-apoptotic granzymes for discrete killing [91, 92]. Instead of pore-forming proteins, macrophages opt for the release of lysosomal cargo to induce heterolysis of cancer cells—likely by severe chemical disruptions [93]. Similar forms of plasma membrane damage can arise from neutrophil extracellular traps, which are web-like structures formed by the release or expulsion of neutrophil contents including antimicrobials and proteases [94–96]. Lastly, neutrophils alongside other immune cells have the propensity to engulf portions of plasma membrane from target cells as a means of intercellular communication (i.e., trogocytosis) [97] or cytotoxicity (i.e., trogoptosis) [98].

### Intracellular

The intracellular environment contains an array of potentially cytotoxic stressors such as ROS, pore-forming proteins, and lysosomal contents, which under normal conditions are regulated by antioxidants, stringent activation requirements, and spatial confinement or zymogens, respectively [54, 99, 100]. When regulation goes awry, physical breaches induced by these stressors can result in, or accompany cell death. Many cell death pathways entail unique forms of plasma membrane damage [99]. In the case of ferroptosis, damage is achieved through iron-dependent lipid peroxidation [15] whereas transmembrane pores are a common feature of necroptosis, secondary necrosis, and pyroptosis [6]. Notably, these pores differ in composition and outcome, as insertion may favor osmotic rupture or weaken plasma membrane resistance against mechanical stress [101–104]. Lastly, the disruption and leakage of lysosomes can lead to detrimental enzyme activity and the onset of lysosomal-dependent cell death [105, 106].

## Plasma membrane repair of physical breaches

Early observations in mechanically injured cells noted the ubiquitous requirement of extracellular calcium for membrane resealing [107–109]. Indeed, the steep calcium gradient between the extra- and intra-cellular environment (~ 2 mM and 100 nM, respectively) ensures that calcium entry is an ideal alarm for membrane damage [110]. Calcium influx through the wound site can be further amplified by voltage-gated channels and internal stores to bolster repair efforts [111, 112]. This leads to local activation of calcium-dependent proteases, such as calpains (CAPN), which promote disassembly of the cytoskeleton through cleavage of substrates including vimentin and cortactin [113, 114]. Alongside relieving membrane tension, this clears the landscape for incoming repair machinery, vesicle fusion events, and cytoskeleton remodeling to restore integrity.

It is convenient to conceptualize the requirements for cell repair by paralleling those of tissue healing, encompassing four complementary stages [115]: hemostasis (sealing off the breach), inflammation (removal of dangerous material that entered the cell), proliferation and migration (replacement of lost or damaged cell components), and remodeling (adaptive responses). In the context of membrane repair, these latter three steps are collectively referred to as “regeneration,” as they represent the steps needed to restore sites of damage to their original state. The means by which cells seal off a physical breach has received far more attention than the other steps of cell repair.

### Sealing off the breach

**Fig. 3:**
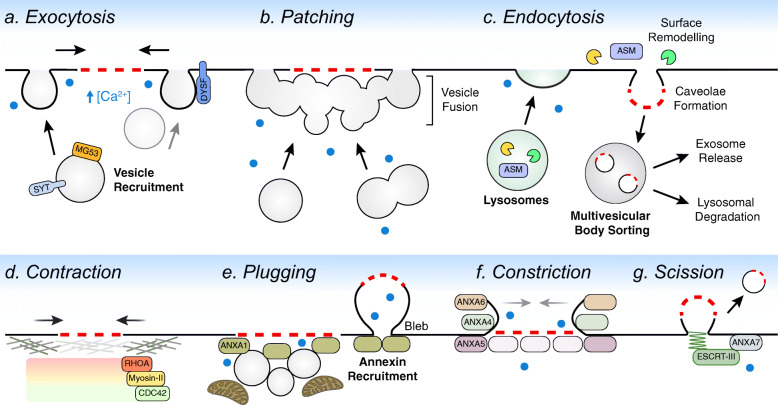
Plasma membrane repair pathways to seal a physical breach. Depending on the extent of damage, several different pathways can cooperatively facilitate wound closure. (a) Exocytosis can relieve membrane tension at the wound site to promote wound closure. Vesicle recruitment to the cell surface occurs through kinesin- and myosin-dependent transport and may require additional support by proteins such as MG53. At the wound site, calcium-dependent fusion machinery such as synaptotagmins (SYT) or dysferlin (DYSF) mediate vesicle fusion. (b) Patching entails inter-vesicle fusion underneath the wound site to generate a membrane patch to seal large tears. (c) Lysosomal exocytosis can promote caveolar endocytosis upon the extracellular release of cathepsins and acid sphingomyelinase (ASM). The fate of endocytosed lesions is determined upon sorting in multivesicular bodies. (d) Concentric zones of actin regulators (e.g., RHOA, myosin-II, CDC42) can form around the wound site to facilitate closure by actomyosin contractions. (e) The accumulation of vesicles, calcium-sensitive proteins (e.g., ANXA1), and mitochondria at the wound site can form a temporary plug to limit diffusion of materials between the extra- and intra-cellular environment. (f) Different annexins can assemble along the wound edge to limit wound expansion (ANXA5), induce membrane curvature (ANXA4), and generate constriction force (ANXA6) to seal a breach. (g) The ANXA7-dependent recruitment of ESCRT-III machinery to the wound site can lead to membrane scission and the release of damaged membrane

While the tiniest of wounds can be healed by spontaneous lipid flow, more substantial injuries such as nanoruptures, tears, and pores require active repair by interior cell constituents. Both the size and nature of the wound (i.e., exposed lipid edge versus pore) are governing factors in mounting an appropriate repair response [28, 34]. As highlighted below, there are several mechanisms by which cells achieve plasma membrane repair (Fig. 3). Importantly, most of these pathways are not mutually exclusive and collaborate to seal off breaches.

#### Exocytosis

Exocytosis has been proposed to promote wound closure by lowering membrane tension to allow for membrane flow over wounds that would otherwise simply gape open [116]. This form of vesicle mediated-repair is calcium-dependent [117] and has been demonstrated to involve the fusion of vesicle compartments with the plasma membrane such as secretory granules [118–120], reserve granules [121], and lysosomes [122]. The importance of exocytosis in resealing is demonstrated by the lack of successful repair following manipulations that impair basic components of the exocytotic fusion machinery such as SNAREs and synaptotagmins (syt) [117, 123].

During repair, lysosomal fusion at the plasma membrane is mainly attributed to syt-VII activity; however, observations during astrocyte repair highlight cell type-specific differences in syt requirements [122, 124]. In the case of muscle repair, lysosomal exocytosis is also dependent on dysferlin, a calcium-dependent membrane-binding protein that can promote lysosome tethering to the cell surface [125, 126]. Dysferlin can be cleaved into a syt-like molecule which could render it able to directly promote fusion of intracellular vesicles with the plasma membrane [127].

Another potential facilitator of exocytosis is MG53, a ubiquitin ligase normally found in the cytosol, plasma membrane, and on intracellular vesicles [128]. Upon damage, MG53 accumulates at the wound site in an oxidation-dependent manner where it is thought to interact with caveolae-related proteins and phosphatidylserine [128–131]. Based on its localization to wound sites, it has been proposed that MG53 could help the recruitment of membranous compartments to facilitate repair [128].

#### Patching

The repair of larger tears has been proposed to occur through “patching,” whereby inter-vesicle fusion forms an underlying patch that fuses along the exposed edge of the wound. Whether integration of this patch at the wound site is directly achieved through exocytosis or fusion upon lipid disorder remains unclear [132]. Patching was initially proposed based on the observation that sea water injected into echinoderm eggs is walled off from the rest of the cytoplasm by what appears to be a membrane in electron micrographs [133]. More recently, patching has been directly observed in wounded *Xenopus* oocytes [120]. Importantly, both echinoderm eggs and *Xenopus* oocytes are unusually large cells and patching involves unique sources of vesicle (e.g., reserve granules and secretory granules) that are unspecified in other cell types. While evidence of patching in mammalian cells is scarce [134], it remains a solution for sealing larger tears given the accumulation of membranous compartments observed at wound sites [109, 128].

#### Endocytosis

The removal of smaller tears and transmembrane pores can be achieved through caveolar endocytosis [135, 136]. This process is facilitated through lysosomal exocytosis leading to the exofacial release of acid sphingomyelinase which can generate ceramide-enriched caveolae that are subsequently internalized [137]. Endocytosed lesions then undergo sorting in multivesicular bodies by members of the endosomal sorting complexes required for transport (ESCRT) family where they are destined for either lysosomal degradation or exosome release [138, 139]. Oxidative stress can also trigger lysosomal exocytosis [140], although whether this similarly triggers the removal of plasma membrane with chemical disruptions is unknown. Furthermore, it remains unclear how endocytosis could seal off larger tears (> 100 nm) which could not be accommodated by individual caveolae (50–100 nm) unless clustering allows for the processing of more extensive wounds [141, 142].

#### Contraction

A physical breach can be sealed through actomyosin contractions by cortical actin filaments (F-actin) and myosin-II that encircle the damage site [143]. Contraction-mediated repair has been observed in mechanically injured *Xenopus* oocytes and embryos [144, 145], *Drosophila* syncytium [146], and *C. elegans* epidermis [147, 148]. In these models, concentric zones of actin regulators (e.g., small GTPases such as RhoA and Cdc42) form around the wound site to facilitate the organization and contractile movement of actin rings [144, 146, 149, 150]. Such contractions have also been observed in transected invertebrate neurons [151], where it is thought to bring the edges of plasma membrane close enough for membrane fusion events to take over [152]. However, this contraction response is relatively slow, making it unlikely to account for those situations in which membrane resealing occurs within several seconds [153]. More broadly, the accumulation of actin and non-muscle myosin IIA at wound sites is critical for the delivery of repair machinery [154–156] and can indirectly promote resealing by controlling bleb dynamics in response to pore-induced damage [157].

#### Plugging

Plugging refers to the aggregation of vesicles and other material (e.g., mitochondria, proteins) at the wound site, forming the single cell equivalent of a clot [158–160]. Plug formation has been observed in response to small and large breaches, where it is proposed to limit exchange of material between the cytoplasm and the extracellular space while other forms of repair proceed. A role for plugging is supported by the accumulation of tightly opposed, membranous compartments at wound sites in essentially all cell types [109, 120, 152, 161]. Both annexins (ANXA) and dysferlin are promising candidates to promote plugging as they are calcium-dependent, membrane-binding proteins recruited to the wound site with the potential to crosslink vesicles to each other or the plasma membrane [110, 111, 120, 162, 163]. Regardless of the mechanism, because plugs fail to restore a uniform membrane at the damage site, they are necessarily temporary.

#### Constriction

Based on their known physical properties, certain annexins can facilitate wound closure through membrane constriction [164]. Upon recruitment to the damage site, trimers of ANXA4 bend the plasma membrane to promote constriction force dependent on ANXA6 [165]. This activity could potentially be assisted by ANXA5 arrays that suppress wound expansion, in addition to vesicle fusion events necessary to provide additional membrane required for constriction-mediated closure [166, 167].

#### Scission

Physical breaches situated on outward buds or extensions of the plasma membrane can be removed by ESCRT complexes [28]. Calcium influx promotes ANXA7-dependent recruitment of ESCRT-III members to the damage site, where they form a multi-subunit contractile lattice that snips off membrane containing a wound [28, 168]. With the exception of large tears (> 100 nm) [28], scission can facilitate the release of smaller breaches including membrane damaged by lipid peroxidation [9, 169–171]. Notably, the outward buds targeted by scission are distinct from larger, plasma membrane blebs that retract through actomyosin contractions [157, 172]. Exactly how the ESCRT-III system can discern between these membrane structures during repair is unclear.

### Removal of dangerous material

Two major unwanted incursions from wounding are elevations in intracellular calcium and oxidation, which, if not terminated, will eventually kill the cell. A major source of calcium removal is through uptake by organelles such as the endoplasmic reticulum and mitochondria [173–175]. In the case of the latter, calcium uptake leads to mitochondrial fragmentation to promote greater calcium clearance and the release of local redox signals critical for repair [176, 177].

Similar to calcium, oxidative species are particularly high in the extracellular environment and enter the cell upon a breach [8]. During repair, cells rely on antioxidants (e.g., vitamin E, glutathione, GPX4) to prevent the onset of lipid peroxidation and irreparable damage, as evident in muscle cells depleted of these defenses [178]. In support of this notion, cells pretreated with lipid-directed antioxidants undergo faster rates of resealing [37, 179]. It remains unclear whether oxidant-induced damage solely exacerbates the initial breach or leads to the formation of new disruptions, which may spread repair efforts thin and lower the rate of successful resealing.

### Replacement of lost or damaged cell components

A physical breach inevitably results in the leakage of cytosolic content into the extracellular environment. The loss of smaller molecules, such as ions and ATP, is common to most forms of breaches whereas larger wounds lead to substantial losses of protein [30, 180, 181]. Recovery of such material has mainly been studied in the context of potassium efflux during pore-induced damage. A decrease in intracellular potassium activates cytoprotective mitogen-associated protein kinase (MAPK) signaling-dependent events that partially restore levels in the subsequent hours following damage [182]. In addition, potassium loss triggers autophagy activation which likely recycles damaged cargo in the vicinity of the wound site [182]. Our current understanding of what seeps through a breach remains limited to only a handful of molecules, and exactly how and when lost material is replenished remains to be determined.

Upon damage, the sudden influx of calcium favors the local disassembly of the actin cytoskeleton, which, while necessary for vesicle fusion, is unfavorable for other steps of repair [154, 183]. Actin polymerization at the wound site is partially achieved upon the recruitment of ANXA2 and its binding partner S100A11, in addition to redox-dependent RhoA activity [7, 159]. These contributors of F-actin are critical for successful repair, which prevents further calcium influx and allows restoration of the actin cortex.

Membrane repair also leads to extensive remodeling of the plasma membrane and extracellular matrix [184]. Vesicle fusion events replenish plasma membrane that is directly lost from wound scission or endocytosis. However, an indirect consequence of removing plasma membrane is the accompanying loss of surface receptors and disruption of microdomains leading to impairments in cell signaling [185, 186].

### Adaptive responses

Scars represent a “memory” of tissue damage; similarly, cells retain a “memory” of initial damage events which serve to enhance repair upon subsequent injury [187]. Such adaptive responses are most well understood in the context of purinergic signaling, whereby ATP leakage into the extracellular environment stimulates purinergic receptors (e.g., P2X7, P2Y) on both damaged and non-damaged cells [188]. This form of signaling triggers calcium influx which can promote cytoprotective blebbing even in non-damaged cells [189]. More importantly, calcium influx activates protein kinase activity which is required for priming vesicle-mediated repair to enhance the rate of resealing upon a second injury [123]. This protective response is evident in both the short-term (i.e., 5-min intervals between injury) [190] and long-term (i.e., 24 h) [191], albeit the latter is dependent on transcriptional changes. Other adaptive responses include tissue growth, as best described in muscle following the leakage of fibroblast growth factor upon exercise-induced damage [192]. Furthermore, cellular priming is not limited to soluble factors as damaged membrane, or vesicles shed during repair can be internalized by nearby cells to initiate signals including proliferation and even macrophage polarization [193–195].

## Plasma membrane damage and repair: whole-body implications

**Fig. 4:**
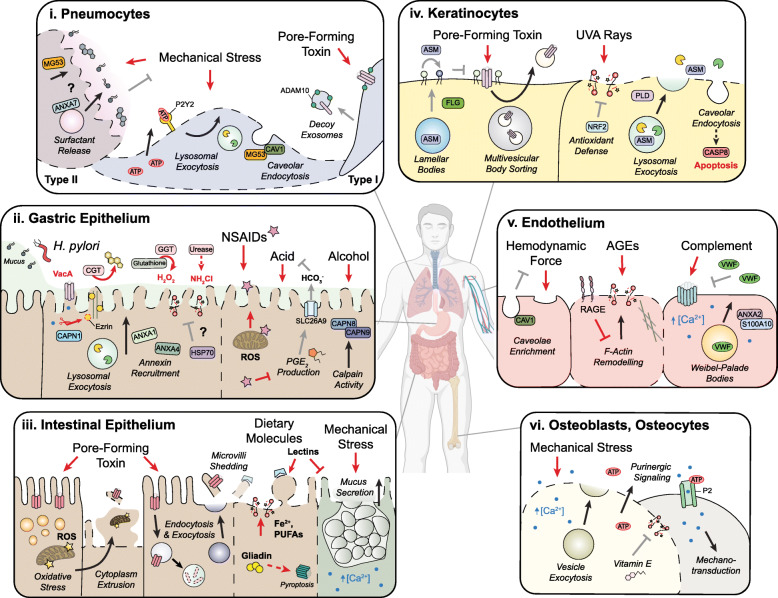
Plasma membrane damage and repair in the lung, gastrointestinal tract, skin, vasculature, and bone. Red arrows: sources of plasma membrane damage; black arrows: repair pathways; gray arrows: forms of cellular resistance. Human body was created with BioRender.com. **(i) Pneumocytes:** Mechanical stress during ventilation is typically alleviated by surfactant. Damage-induced ATP leakage promotes lysosomal exocytosis via P2Y2 receptors. MG53 facilitates repair in type I cells through caveolar endocytosis, although its protective role in type II cells remains unclear. Type II cells likely facilitate resealing through ANXA7-dependent fusion of surfactant granules. During S. aureus infection, pneumocytes evade damage from pore-forming toxin by releasing decoy exosomes enriched in host receptor ADAM10. **(ii) Gastric Epithelium:** Mucus integrity is compromised during H. pylori infection and by amphiphilic molecules such as NSAIDs and alcohol. Pore formation by VacA disrupts microvilli organization upon CAPN1-mediated cleavage of ezrin. Cholesterol extraction and lipid peroxidation are achieved by virulence factors including cholesterol-α-glucoside transferase (CGT), γ-glutamyl transpeptidase (GGT), and urease (via monochloramine, NH4Cl). Gastric repair includes lysosomal exocytosis and annexins, whereas HSP70 activity alleviates chemical disruptions although its exact role remains unclear. Meanwhile, NSAIDs and alcohol elicit damage through direct interactions with plasma membrane phospholipids or indirectly via oxidative stress. Cytoprotective factors include calpains and prostaglandin E2 (PGE2), the latter of which stimulates bicarbonate (HCO3-) release via SLC26A9 to alleviate acid-induced injury. **(iii) Intestinal Epithelium:** Enterocytes rid bacterial pore-forming toxins (~ 1–2 nm) through cytoplasm extrusion, preceded by oxidative stress as evident by lipid droplet formation and mitochondrial damage. Pores are also removed through vesicle trafficking events and microvilli shedding. Dietary lectins can lead to microvilli abnormalities and inhibit mucus secretion in goblet cells which is a form of membrane resealing. Other dietary molecules, such as poly-unsaturated fatty acids (PUFA) and undigested gliadin peptide, can promote damage through lipid peroxidation and pyroptosis, respectively. **(iv) Keratinocytes:** During S. aureus infection, keratinocytes internalize α-toxin pores and release them via exosomes. Resistance is achieved through the filaggrin (FLG)-dependent release of acid sphingomyelinase to reduce the availability of exofacial sphingomyelin, an alternative receptor of α-toxin. Ultraviolet A irradiation causes lipid peroxidation that is alleviated by NRF2-dependent antioxidant defenses. Phospholipase D (PLD) activity promotes vesicle fusion events such as lysosomal exocytosis. Alongside repair, caveolar endocytosis can result in caspase-8-mediated apoptosis. **(v) Endothelium:** Endothelial cells buffer hemodynamic force through caveolae. Advanced glycation end products (AGEs) entice lipid peroxidation whereas overexpression of receptor for AGEs (RAGE) prevents F-actin remodeling required for resealing. Complement-induced damage triggers the release of von Willebrand factor (VWF) which can limit further complement deposition. **(vi) Osteoblasts, Osteocytes:** Bone cells experience nanoruptures during locomotion that can be repaired through exocytosis with an apparent role for dietary Vitamin E in limiting further oxidative damage. ATP leakage from the wound site initiates calcium-dependent mechanotransduction in nearby, uninjured cells through P2 receptors

**Fig. 5:**
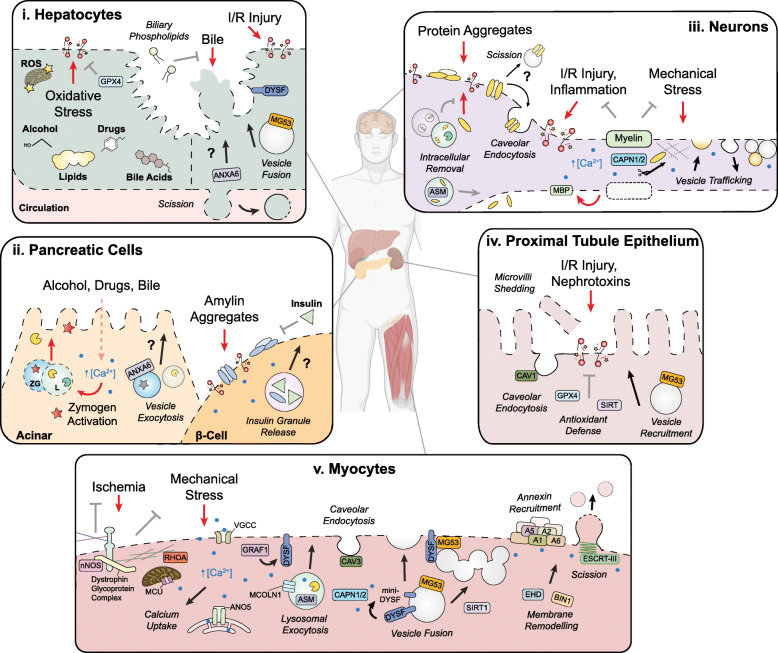
Plasma membrane damage and repair in the liver, pancreas, nervous system, kidney, and muscle. Red arrows: sources of plasma membrane damage; black arrows: repair pathways; gray arrows: forms of cellular resistance. Human body was created with BioRender.com. **(i) Hepatocytes:** Alcohol and drug metabolism or the accumulation of lipids and bile acids can promote lipid peroxidation which is alleviated by glutathione peroxidase 4 (GPX4) activity. Basolateral wounds can be removed through membrane scission whereas apical protrusions are prone to rupture. Biliary phospholipids confer protection by reducing the ability of bile acids to solubilize membrane. Ischemic-reperfusion injury (I/R injury) triggers dysferlin-mediated exocytosis which may involve ANXA6 activity given its role in hepatocyte vesicle trafficking. **(ii) Pancreatic Cells:** Acinar cell damage can indirectly arise following exposure to stressors such as alcohol, drugs, and bile. Abnormally high levels of intracellular calcium prompt the fusion of zymogen granules (ZG) with lysosomes (L), leading to the premature activation of zymogens (e.g., trypsin) which inflict membrane damage upon leakage into the cytosol. Pancreatic β cells experience membrane damage from amylin aggregates in the extracellular environment that are typically prevented by insulin co-secretion. In both cell types, repair likely involves exocytosis based on the abundance of granules and lysosomes underlying the plasma membrane. **(iii) Neurons:** Neuronal membrane damage can arise from exposure to protein aggregates (e.g., β-amyloid) in the extra- or intra-cellular environment which elicit mechanical damage and oxidative stress. Depending on the protein, resistance against intracellular aggregation may be achieved through multivesicular body sorting and lysosomal degradation or exocytosis. Lesions from β-amyloid aggregates may be removed through caveolar endocytosis and ESCRT-III activity as observed in other cell types. Oxidative stress can lead to nanoruptures in axonal membrane which is inherently protected by myelin sheath. Demyelination can exacerbate membrane damage upon the release of myelin basic protein (MBP). Neuronal repair entails calpain activity and vesicle trafficking events such as exocytosis, endocytosis, and plugging. **(iv) Proximal Tubule Epithelium:** Renal cells experience lipid peroxidation during I/R injury and exposure to nephrotoxins which are alleviated by antioxidants such as GPX4 and sirtuins (SIRT). Physical breaches are repaired through membrane remodeling events including microvilli shedding, caveolar endocytosis and MG53-mediated vesicle recruitment. **(v) Myocytes:** Mechanical stress is buffered through the dystrophin glycoprotein complex which connects the extracellular matrix to the actin cortex. This complex also anchors nitric oxide synthase (nNOS) at the cell surface to prevent ischemic injury. Upon damage, calcium influx is amplified by voltage-gated calcium channels (VGCC) and the release of lysosomal stores (MCOLN1). Calcium uptake required for successful repair is achieved by the endoplasmic reticulum and mitochondria, the latter of which promotes redox-dependent RhoA activity to drive F-actin assembly. GRAF1 promotes dysferlin at the plasma membrane where it can facilitate lysosomal exocytosis and patching. Vesicle fusion can also be achieved upon the calpain-dependent cleavage of dysferlin into a syt-like molecule. Vesicle recruitment to the wound site is promoted by MG53 and SIRT1 activity. Annexins (A) also promote wound closure by forming a highly organized repair cap which may drive constriction. Membrane remodeling is further achieved by the recruitment of regulators, such as EHD and BIN1, in addition to ESCRT-III-mediated scission

In vivo, cellular architecture and composition are adapted to minimize plasma membrane damage by tissue-specific stressors. For example, muscle and endothelial cells have an abundance of caveolae to buffer frequent mechanical stress [196, 197] whereas metabolically active cells such as hepatocytes mitigate chemical disruptions through antioxidant defenses [198]. These forms of resistance are often compromised amidst disease and render the plasma membrane susceptible to damage (Table 1). While membrane repair pathways are generally conserved, cell type-specific adaptations arise from inherent differences in cell structure and the expression of repair machinery. Furthermore, genetic factors can have tissue-specific impacts on plasma membrane resistance and repair that may be causative of or exacerbate disease (Table 2). Here, we highlight our current understanding of cell type-specific damage and repair throughout the body (Figs. 4 and 5).

**Table 1:** Tissue-specific stressors of plasma membrane integrity

Tissue	Source	Stressor	Injury or disease	Refs
**Lung**	Mechanical	Ventilation	Ventilator-induced lung injury	[199]
	Chemical	Tobacco	COPD	[200, 201]
		Asbestos	Asbestosis	[202, 203]
	Microbial	Pore-forming toxin	Bacterial pneumonia	[204–206]
**Oral cavity**	Mechanical	Tooth movement, brushing	–	[207, 208]
	Chemical	Tooth whitening agents Tobacco	-- Oral lesions	[209]
				[210, 211]
**Esophagus**	Chemical	Gastric acid	Gastroesophageal reflux disease	[212]
	Intracellular	ROS		[213]
**Stomach**	Chemical	Gastric acid, NSAIDs, alcohol	Peptic ulcers, gastritis	[23, 214]
	Microbial	Pore-forming toxin, cholesterol transferase	*Helicobacter pylori* infection	[215]
**Intestine**	Chemical	Dietary lectins, iron	–	[216, 217]
		NSAIDs, bile	Ulcers	[69]
	Microbial	Pore-forming toxin	Food-borne illness, IBD	[13, 182]
	Intracellular	Oxidative stress	Necrotizing enterocolitis, IBD	[19, 218]
**Skin**	Mechanical	Locomotion	–	[2]
		Pruritus (itch)	Inflammatory skin diseases	[219]
	Chemical	Ultraviolet A radiation	–	[220]
	Microbial	Pore-forming toxin	Atopic dermatitis	[221]
**Vasculature**	Mechanical	Cardiac output	–	[197]
	Chemical	AGEs	Type 1 and type 2 diabetes	[222, 223]
		β-amyloid	Alzheimer’s disease	[224]
	Immune	Complement	Von Willebrand disease	[225]
	Microbial	Pore-forming toxin	Sepsis	[226]
**Bone**	Mechanical	Physical loading	–	[26, 179]
**Liver**	Chemical	Drugs, alcohol, lipid accumulation	Acute liver failure, alcoholic liver disease, NAFLD	[227]
		Bile acids	Cholestasis, familial intrahepatic cholestasis	[228, 322]
	Microbial	Pore-forming toxin	Listeriosis	[70]
**Pancreas**	Mechanical	Amylin	Type 2 diabetes	[229]
	Intracellular	Zymogen activation	Acute pancreatitis	[230]
**Nervous system**	Mechanical, Chemical	Protein aggregates	Neurodegenerative diseases	[231]
	Chemical	I/R injury	Stroke	[232]
	Immune	Oxidative stress	Multiple sclerosis, AE	[27, 233]
**Kidney**	Chemical	I/R injury, nephrotoxins	Acute kidney injury	[234]
**Muscle**	Mechanical	Eccentric contraction	Muscular dystrophies, Niemann-Pick type A/B disease	[11, 235]
	Chemical	I/R injury	Duchenne’s muscular dystrophy	[236]
		AGEs	Type 1 and type 2 diabetes	[237]
		Cardiotoxin	Snakebite	[21]
**Heart**	Mechanical	Cardiac output	Cardiomyopathies	[238]
	Microbial	Viral protease	Viral myocarditis	[239]

*COPD*, chronic obstructive pulmonary disease; *ROS*, reactive oxygen species; *NSAIDs*, non-steroidal anti-inflammatory drugs; *IBD*, inflammatory bowel disease; *AGEs*, advanced-glycation end products; *NAFLD*, non-alcoholic fatty liver disease; *I/R*, ischemia-reperfusion; *AE*, autoimmune encephalomyelitis

**Table 2:** Disease-associated factors that impact plasma membrane resistance and repair

Impact	Protein	Disease	Refs
**Lung**			
Resistance, repair	CAV1, MG53	Idiopathic pulmonary fibrosis	[240, 241]
**Gastrointestinal tract**			
Resistance, repair	CAPN8, CAPN9	Gastropathies	[242]
Repair	ANXA4	Gastric cancer	[243]
	ATG16L1	Inflammatory bowel disease	[13]
	GPX4	Inflammatory bowel disease	[19]
**Skin**			
Resistance	FLG	Atopic dermatitis	[221]
**Vasculature**			
Repair	VWF	Von Willebrand disease	[225]
	ANXA2	Behcet’s disease	[244]
**Liver**			
Resistance	MDR3	Familial intrahepatic cholestasis	[228]
**Kidney**			
Repair	MG53	Acute kidney injury	[245]
**Muscle**			
Resistance	DMD	Duchenne’s muscular dystrophy	[246]
	DAG1	Dystroglycanopathy	[247]
Repair	DYSF	Limb-girdle muscular dystrophy type 2B	[125]
	CAV3	Limb-girdle muscular dystrophy type 1C	[196]
	ANO5	Limb-girdle muscular dystrophy type 2 L	[173]
	PTRF	Lipodystrophy	[248]
	MICU1	Neuromuscular disorder	[174]
	ANXA6	Limb-girdle muscular dystrophy type 2C	[249]
	ASM	Niemann-Pick type A/B	[235]
	CAPN-1, −2, −3	Calpainopathies	[34, 113, 250]
	MCOLN1	Mucolipidosis type IV	[112]
	SYT7	Autoimmune myositis	[251]
	HRK	Myositis	[252]
**Heart**			
Repair	DYSF	Limb-girdle muscular dystrophy type 2B	[238]
	MG53	Valvular heart disease	[253]

### Lung

The alveolar epithelium is comprised of type 1 pneumocytes which control gas exchange and are replenished by surfactant-producing type 2 pneumocytes. During normal respiration, alveolar cells experience mechanical stress that is alleviated by surfactant production, basement membrane elasticity, and an abundance of caveolae on type I cells [240, 254, 255]. Exceedingly high tidal volumes (e.g., ventilation therapy) can result in mechanical damage as observed in ventilator-induced lung injury [199, 256]. This type of mechanical injury is exacerbated by dysfunctional surfactant, common to several disorders including acute respiratory distress syndrome and pulmonary fibrosis [257].

In type 1 pneumocytes, mechanical injury leads to actin depolymerization and endomembrane recruitment [258]. Lysosomal exocytosis partially accounts for these vesicle fusion events, and in pneumocytes, this process is dependent on purinergic signaling [259]. Caveolar endocytosis, promoted by MG53 activity, is another prominent form of repair in type I cells [240, 260] and while MG53 also fosters resealing in type II cells, its role in this context remains unclear [261]. Abnormalities in caveolae may accelerate ventilator-induced damage in pulmonary fibrosis patients who display reduced levels of caveolin-1 and mislocalization of MG53 [241, 261]. It is foreseeable that functional differences between these two cell types, such as surfactant secretion, may influence their resistance to injury and repair capacity. For instance, type II cells may trigger the release of specialized surfactant granules given this is a Ca^2+^-dependent process dependent on repair machinery such as ANXA7 [262, 263].

Pneumocytes are also exposed to microbial and chemical insults following inhalation. During bacterial respiratory infections, pathogenic microbes produce pore-forming toxins that elicit pneumocyte death by direct lysis or indirectly via necroptosis [204, 205]. In the case of smaller pores, such as α-toxin, pneumocytes generate decoy exosomes enriched in toxin receptor to circumvent membrane insult [206]. Meanwhile, the inhalation of inert particles (e.g., asbestos) and cigarette smoke can promote pneumocyte damage by lipid peroxidation [200–203].

### Upper digestive tract

Cells within the oral cavity, such as the gingival epithelium surrounding teeth, can experience mechanical damage from orthodontic tooth movement and brushing [207, 208]. Chemical disruptions can arise from tooth whitening agents and lead to damaging oxidative stress in periodontal ligament cells [209]. Similarly, exposure to cigarette smoke and moist smokeless tobacco—known inducers of oral lesions—appear to induce both mechanical damage and lipid peroxidation, the latter of which is detectable in the saliva of smokers [210, 211].

Further down the digestive tract, the esophageal epithelium is susceptible to damage from contact with refluxed stomach contents during gastroesophageal reflux disease [264, 265]. Early studies demonstrated exposure of esophageal tissue to stomach acid and pepsin leads to intracellular acidification and the onset of cell necrosis [212, 266]. Exactly how cell acidification results in a physical breach remains elusive. Alternatively, acid and bile exposure can provoke ROS and cytokine production to create a damaging inflammatory milieu [213, 264]. Both mechanisms likely result in membrane damage through lipid peroxidation, considering the ability of lipid-directed antioxidants to ameliorate reflux severity in vivo [267]. Repair defects in the esophageal epithelium may contribute to the pathogenesis of Barrett’s esophagus which arises from prolonged damage [268].

### Stomach

Mucus lining the gastrointestinal epithelium provides physical and biochemical protection against extracellular stressors [269]. Weakening of this mucus layer often precedes gastric cell injury during *Helicobacter pylori* infection and frequent NSAID exposure [23, 270]. Upon achieving close contact with the apical membrane of gastric cells, *H. pylori* inflicts damage through an arsenal of virulence factors [215]. Physical breaches caused by the pore-forming toxin, VacA, can disrupt microvilli and the function of acid-secreting Parietal cells [271, 272]. Alternatively, *H. pylori* effectors can induce chemical disruptions such as cholesterol extraction [273] and lipid peroxidation through the formation of monochloramine, a lipophilic oxidant [274]. Accordingly, heightened levels of lipid peroxidation are observed in gastric biopsies from *H. pylori*-infected patients and such damage is exacerbated by the bacterial-induced degradation of host glutathione [275, 276]. During *H. pylori* infection, membrane repair events such as annexin recruitment and lysosomal exocytosis have been observed, with an apparent role for HSP70 in alleviating chemical disruptions [243, 274]. Intriguingly, the overexpression of ANXA4 in *H. pylori*-associated gastric tumors may confer a survival advantage through enhanced resealing efforts [243, 277].

Non-steroidal anti-inflammatory drugs (e.g., aspirin) are commonly used for relieving inflammation and pain; however, frequent usage is associated with gastrointestinal lesions (e.g., peptic ulcers) [278]. Chemical disruptions can arise from direct interactions with plasma membrane phospholipids or as a consequence of mitochondrial dysfunction [279, 280]. NSAIDs can further exacerbate injury by inhibiting cyclooxygenase-dependent production of prostaglandin E_2_ (PGE2), a cytoprotective lipid mediator against gastric tissue damage [281, 282]. While its role in plasma membrane integrity remains elusive, PGE2 may enhance lysosomal exocytosis or alleviate acid-induced damage as previously described [283, 284].

Alcohol intake is another causative agent of gastritis and similar to NSAIDs, ethanol can compromise mucus integrity and promote lipid peroxidation [214, 285]. Early studies demonstrated the ability of absolute ethanol to cause gastric cell necrosis, presumably through extensive chemical disruptions [63, 281]. Rapid mucus secretion was observed in this injury model and later demonstrated to be an intrinsic response to membrane damage in surface mucus cells [63, 119]. In this context, repair may be achieved by gastric-specific calpains as mice deficient in CAPN8 or CAPN9 are more susceptible to ethanol-induced injury [242]. However, damage was only assessed on a histological level so the role of these calpains in gastric cell plasma membrane integrity remains unclear. Intriguingly, the authors highlighted missense variants in CAPN8 and CAPN9 that correspond to pathogenic mutations in CAPN3, which is relevant to muscular dystrophy [242, 250]. This raises the notion that genetic defects influencing gastric cell membrane integrity may similarly underlie gastropathies.

### Intestine

The intestinal epithelium persists in a harsh environment and experiences membrane damage even at resting state [286]. Two major sources of damage include pathogenic microbes and ingested chemical stressors [182, 287]. Several enteric pathogens induce intestinal damage through pore-forming toxins that target apical microvilli and intercellular junctions [288, 289]. In *C. elegans*, enterocytes remove small pores (~ 1–2 nm) through endocytosis and microvilli shedding driven by exocytic events [288]. This leads to extensive disruptions in the underlying network of intermediate filaments, which serve to provide resistance against both chemical and mechanical stress [290]. Similar pore-induced damage in *D. melanogaster* enterocytes causes cytoplasm extrusion resulting in cell thinning, and this response is conserved in mammalian cells [291]. Exactly how such small breaches trigger this dramatic repair response is unclear, but oxidative stress is one potential candidate [182, 291].

In the small intestine, NSAIDs such as indomethacin can promote oxidative stress leading to plasma membrane tears and ulceration in vivo [292]. Similar to the stomach, prolonged NSAID exposure dampens intestinal mucus integrity which promotes enterocyte exposure to luminal aggressors including bile and bacteria [23, 69]. It appears that cells at the tips of villi are particularly susceptible to NSAID-induced lipid peroxidation [293] which implies inherent differences in epithelial resistance along the villus-crypt axis.

Dietary molecules, such as lectins, can lead to structural abnormalities in apical microvilli that are self-limiting [216, 294]. Distinct from pore-induced damage, lectin exposure appears to alter membrane fluidity and promote the formation of nanoruptures [295, 296]. Lectins also inhibit damage-induced mucus secretion in Goblet cells, which is a form of exocytosis-mediated repair [297]. Dietary molecules can also trigger membrane damage through indirect mechanisms as observed in celiac disease—certain peptides of gliadin, a driver of the disease, activate pyroptosis and the onset of pore-induced damage [298]. Meanwhile, excessive intake of iron and poly-unsaturated fatty acids can accelerate intestinal lipid peroxidation, the latter of which is particularly relevant to IBD [19, 217].

### Skin

Skin is comprised of cellular diverse layers that form a barrier to the outside environment. Keratinocytes, which are abundant in the outermost layer known as the epidermis, experience mechanical damage during locomotion and pruritus (itch), the latter of which is common to many skin disorders [2, 219]. Exposed to a non-sterile environment, pathogens such as *Staphylococcus aureus* can directly inflict keratinocyte damage through α-toxin pores which are removed by endocytosis and subsequent release through exosomes [139]. Intriguingly, mature keratinocytes achieve resistance against pore-induced injury by secreting sphingomyelinase via lamellar bodies to limit the availability of α-toxin receptor, a strategy disrupted in atopic dermatitis patients with mutations in filaggrin (FLG) [221]. In the case of chronic intracellular *S. aureus* infections, keratinocytes are susceptible to lethal damage from the complement system to rid persistent bacteria by eliminating the host cell—highlighting a case where membrane damage is for the greater good [299].

Ultraviolet A (UVA) exposure from sunlight is a prominent chemical stressor of skin that promotes lipid peroxidation and in extreme cases, ferroptosis [300, 301]. Cell type-specific differences in the extent of UVA damage are highly dependent on intracellular levels of iron, as evident by dermal fibroblasts which are more prone to necrosis than keratinocytes [220]. Keratinocyte resistance against lipid peroxidation is also a reflection of NRF2 activity, a transcriptional regulator of antioxidants important for tissue repair in skin [302, 303]. In response to damaging UVA exposure, keratinocytes undergo lysosomal exocytosis to achieve membrane resealing [304]. In this case, however, the extracellular release of cathepsins was found to trigger caspase-8-dependent apoptosis which may serve to eliminate damaged cells in a discrete manner rather than lytic cell death. The importance of exocytosis for keratinocyte repair is supported by the involvement of phospholipase D, a regulator of vesicle fusion, in resealing mechanical wounds [305].

### Vasculature

Endothelial cells line the vascular network and experience hemodynamic stress during increased cardiac output that can elicit damage, if not for caveolae [197, 306]. Stressors in circulation, such as bacterial pore-forming toxins and host complement, are a prominent source of membrane injury and sustained damage can lead to vascular dysfunction [226, 307]. In type 2 diabetes, hyperglycemia stimulates the formation of advanced glycation end products (AGEs) which can react with phospholipids to stimulate membrane oxidation [308]. Furthermore, AGEs can compromise endothelial repair through F-actin dysregulation, albeit indirectly, via the receptor for AGEs (RAGE) [237, 309]. The interspersed nature of the vasculature system also exposes the endothelium to tissue-specific stressors. For instance in Alzheimer’s disease, β-amyloid peptide can associate with endothelial membrane to elicit neurovascular damage with an emerging role for AGEs in antagonizing this process [222, 224, 310].

In response to complement-induced damage, endothelial cells facilitate repair through the calcium-dependent fusion of Weibel-Palade bodies (WPB) involving ANXA2 [225, 311–313]. The mobilization and fusion of these granules is critical for resealing as damage is exacerbated in cells from von Willebrand disease patients lacking WPBs [225]. Alongside relieving membrane tension, WPB fusion promotes the extracellular release of the clotting protein von Willebrand factor (VWF) to protect against further injury by complement [314]. Defects in this form of repair may contribute to vascular damage in Behcet’s disease considering the presence of ANXA2 autoantibodies [244, 315].

### Bone

Bone formation is attributed to osteoblasts which eventually transition to signal-transducing osteocytes within mineralized tissue. During physiological loading or injury, mechanotransduction promotes bone remodeling through intercellular calcium waves which can be initiated by single plasma membrane damage events [26, 179, 316]. In weight-bearing bones, osteoblasts and osteocytes experience mechanical damage that triggers purinergic-dependent signaling and calcium uptake in nearby, non-wounded cells to stimulate biochemical signals [26, 179]. These lesions are predicted to be nanoruptures (~ 5 nm) based on the leakage of intracellular content and minimal requirement for calpain-dependent repair [26, 34, 179]. During aging, bone becomes less mechanoresponsive, and recent evidence suggests this is due to survival-based selection of osteocytes that display enhanced membrane repair [317].

Despite a clear role for damage in bone mechanotransduction, little is known about plasma membrane repair in these cell types. In osteoblasts, lesions are repaired through protein kinase-dependent exocytic events [26]; however, the identity of such vesicles and how their fusion facilitates wound closure is unknown. Similar forms of vesicle-mediated repair likely occur in osteocytes, which may also shed microvesicles as observed in response to mechanical deformation [318]. Furthermore, antioxidants have a defining role in promoting osteocyte repair as vitamin E-deficient mice display a higher frequency of damage events following acute exercise [319]..

### Liver

Hepatocytes mediate metabolism and detoxification, both of which are energetically demanding processes that generate ROS. Under normal conditions, liver redox homeostasis is critical to prevent the onset of lipid peroxidation, as evident by the premature death of mice lacking GPX4 in hepatocytes [198]. Hepatocytes encounter a multitude of stressors that can promote damaging oxidative stress and contribute to liver disease [227]. For instance in alcoholic liver disease, ethanol can promote lipid peroxidation [320] whereas in non-alcoholic fatty liver disease, intracellular lipid accumulation instigates ROS production [321]. Depending on their concentration, bile acids can elicit hepatocyte membrane damage either directly by solubilizing lipids or indirectly through oxidative stress [322]. These forms of damage are prominent during cholestasis (i.e., bile acid accumulation) which can arise from biliary duct obstruction or impairments in bile acid homoeostasis [323]. Such impairments can be genetically linked as evident by mutations in MDR3, a translocator of biliary phospholipids which prevent the cytotoxic association of bile acids with the plasma membrane [228].

Our understanding of how hepatocytes facilitate resealing is limited. In response to damaging oxidative stress, hepatocytes form membrane protrusions on both apical and basolateral membrane [324, 325]. In vivo imaging revealed that basolateral structures can be released into circulation whereas apical blebs tend to rupture and indicate irreversible cell death. These forms of membrane remodeling likely coincide with dysferlin (DYSF)-dependent vesicle fusion events [326]. Annexin-mediated repair in hepatocytes is enticing given that ANXA6 comprises ~ 0.25% of total hepatic protein and facilitates apical vesicle trafficking [327]. These findings highlight an important role for hepatocyte repair against chemical damage although much remains to be determined.

### Pancreas

The pancreas is an exocrine gland comprised of acinar and β cells which secrete zymogen and insulin granules, respectively. In acinar cells, physiological signals lead to zymogen granule fusion through the controlled release of calcium from internal stores [328]. However, abnormally high levels of intracellular calcium can lead to the premature activation of zymogens (e.g., trypsin) that leak into the cytosol and elicit damage. This form of acinar cell destruction encompasses acute pancreatitis (AP), and major initiators include alcohol abuse and biliary obstruction [328, 329]. These classical stressors promote cytotoxic calcium levels at the expense of internal calcium stores and/or activating plasma membrane calcium channels [330]. While plasma membrane damage can be observed at early timepoints of experimental AP [331, 332], in vitro evidence suggests that plasma membrane damage is an effect, rather than a cause of zymogen activation [230]. Nonetheless, acinar cells likely employ exocytosis-mediated repair given the abundance of lysosomes and secretory granules underlying the apical membrane and the involvement of ANXA6 in vesicle release [333].

Alongside insulin, amylin is released through β cell granules where it regulates glycemic control. In type 2 diabetes patients, there is a link between amylin aggregation and reduced β cells [334, 335]. Amylin is an intrinsically disordered protein with the propensity to aggregate into fibrils that can physically breach the plasma membrane and promote damaging oxidative stress [336–338]. Resistance against this form of injury is partially achieved through the co-secretion of insulin in much higher amounts to prevent amylin aggregation [229]. In contrast, other factors in the microenvironment such as dietary lipids can accelerate fibril formation and exacerbate damage [339, 340]. Considering apoptosis is the leading cause of β cell death [341], amylin-induced membrane damage may stimulate apoptotic cues such as cytotoxic calcium influx and CAPN2 hyperactivation [342, 343]. Membrane repair in β cells has yet to be studied, although granule exocytosis would be consistent with resealing in other secretory cell types [119].

### Nervous system

While the nervous system is comprised of many different cell types, plasma membrane damage and repair are best described in neurons. Neurons transmit signals through 3 structurally diverse parts of the cell: dendrites, the cell body (i.e., soma), and myelin-sheath coated axons. Axonal damage is prevalent in a broad range of central nervous system disorders [344]. During traumatic brain injury, physical trauma can compromise axonal membrane, particularly in superficial layers of the brain [12]. If such trauma disrupts the blood-brain barrier or leads to occlusion (e.g., stroke), neurons are susceptible to secondary injuries such as lipid peroxidation during ischemia [232, 345]. Damaging levels of oxidative stress are suspected to cause nanoruptures in axons within neuroinflammatory lesions, common to multiple sclerosis and autoimmune encephalomyelitis [27, 233]. Axonal membrane resistance to mechanical and chemical damage is partially achieved through myelin sheath, as demyelinated axons or regions inherently exposed (e.g., Nodes of Ranvier) are more prone to injury [27, 233, 346]. Demyelination, common to several neurodegenerative disorders, can further exacerbate injury upon the release of myelin basic protein which has the propensity to breach neuronal membrane [347].

Protein aggregates are a commonality of several neurodegenerative disorders including Alzheimer’s (β-amyloid), Parkinson’s (α-synuclein), and Creutzfeldt-Jakob disease (prion) [231]. Upon processing or misfolding, these intrinsically disordered proteins can form aggregates in the extra- and intra-cellular environment that elicit mechanical damage (e.g., lipid extraction, pore-formation) and promote lipid peroxidation [61, 348, 349]. Neuronal resistance against intracellular aggregate formation is partially achieved through multivesicular body sorting, lysosomal degradation, and even lysosomal exocytosis [350–352]. In the case of β-amyloid, it appears aggregate-induced damage elicits a similar repair response as pore-forming toxins (e.g., caveolar endocytosis, ESCRT activity); however, confirmatory studies in neurons are required [353, 354]. While there is evidence to suggest plasma membrane composition is altered in neurodegenerative disease and through aging [355–357], whether such alterations impact neuronal resistance and repair against protein aggregate-induced damage remains to be determined.

Traditionally, axonal membrane repair has been studied following transection and is extensively reviewed elsewhere [358, 359]. In brief, calcium influx through the wound site is amplified by intracellular stores and voltage-gated calcium channels that lead to calpain activation and the recruitment of vesicles through microtubule transport [358, 360, 361]. At the wound site, immediate closure can be achieved through membrane-based plugs, whereas vesicle fusion events and contraction eventually lead to permanent wound closure [158, 358].

### Kidney

The kidney facilitates blood filtration through renal epithelium organized into functional units termed nephrons that are further subdivided based on role. The proximal tubule is the main site of reabsorption and features high levels of oxidative metabolism to fuel transporter activity [362]. Consequently, the proximal tubule is a common site of damaging oxidative stress arising from I/R injury or exposure to nephrotoxins (e.g., chemotherapeutics) [362]. Proximal tubule cell injury encompasses many forms of acute kidney injuries (AKI) that can lead to chronic kidney disease [234]. Depending on location along the proximal tubule (i.e., segments 1–3), renal cells display differences in their resistance to chemical damage. For instance, ischemic damage leads to extensive necrosis of segment 3 whereas cells in the preceding segments repair successfully [55]. Such differences in membrane resistance are partially attributed to antioxidant production, and in the case of segment 1 cells, the absence of ROS-producing peroxisomes [363, 364].

In vivo, renal lipid peroxidation is actively repaired by GPX4 to prevent ferroptosis and the onset of AKI [365]. Renal protection against oxidative damage is also achieved by members of the sirtuin family (SIRT), a group of NAD^+^-dependent deacetylases that regulate redox signaling. For instance in response to cisplatin-induced AKI, SIRT1 and SIRT3 can alleviate oxidative stress generated by peroxisomes and mitochondria, respectively [366, 367]. Upon ischemic injury, proximal cells undergo extensive membrane remodeling events including caveolar endocytosis and microvilli shedding [55, 368]. These forms of repair may be facilitated by MG53 which was found to be protective against AKI in mice [245].

### Muscle

During muscle function, myocytes buffer mechanical stress by stabilizing the plasma membrane (i.e., sarcolemma) through the dystrophin glycoprotein complex in addition to an abundance of caveolae [11, 196]. However, eccentric muscle contractions can lead to physical breaches in the sarcolemma that require active repair [369]. Genetic abnormalities in either myocyte resistance or repair encompass myopathies characterized by progressive muscle weakening, collectively referred to as muscular dystrophy (MD) [11]. Myocyte resistance against mechanical stress is weakened by mutations in dystrophin and dystroglycan that disrupt association of the sarcolemma with the cytoskeleton and extracellular matrix, respectively [246, 247]. In the case of Duchenne’s MD, dystrophin deficiency can indirectly result in ischemic damage due to the absence of nitric oxide activity at the cell surface to promote vasodilation [236]. Alternatively, other forms of MD can weaken myocyte resistance through defects in caveolae formation and improper muscle regeneration [250, 370].

Myocyte repair has been extensively studied in physiology and disease [371]. A physical breach in the sarcolemma leads to a rise in intracellular calcium due to entry through the wound site, voltage-gated calcium channels (VGCC), and lysosomal release through MCOLN1 [111, 112]. Cytotoxic calcium levels are partially prevented by organelle uptake as observed in mitochondria and endoplasmic reticulum via the mitochondrial calcium uniporter (MCU) and anoctamin-5 (ANO5), respectively [173, 174]. Calcium influx activates CAPN1 and CAPN2 activity essential for resealing tears in muscle [34, 127]. Dysferlin mediates vesicle fusion events in addition to promoting lysosomal exocytosis, and genetic abnormalities in this factor underlie defective repair in limb-girdle MD type 2B [125, 126]. Other potential regulators of exocytosis-mediated repair include GRAF1 and SIRT1, although the influence of the latter on vesicle dynamics remains unclear [372, 373]. Sarcolemma resealing is also facilitated by ESCRT-III activity and annexin recruitment, whereby annexins form an actin-dependent structure termed a repair cap [167, 374, 375]. Based on electron micrographs of this structure [161], the cap is likely equivalent to the complex network of membrane protrusions seen at wounds in other cell types [109, 153]. The accumulation of MG53, dysferlin, EHD1, and BIN1 adjacent to the repair cap suggests simultaneous vesicle fusion events and membrane remodeling to relieve tension during annexin-mediated closure [167, 376]. Intriguingly, a genetic variant of ANXA6 was identified as a modifier of MD in part through its ability to disrupt dysferlin recruitment to the repair cap [249, 377]. This reveals that inherent defects in plasma membrane repair can be polygenic in nature.

### Heart

The heart is under continuous mechanical stress that potentiates plasma membrane damage in cardiomyocytes, aortic endothelium, and valve interstitial cells [3, 253, 306]. Following intensive cardiac output, membrane repair is critical to prevent the onset of heart pathologies such as cardiac arrest and ventricular injuries [238, 378]. As in skeletal muscle, cardiomyocyte resealing is dependent on calpain activity and dysferlin-dependent vesicle fusion [238, 378]. Defects in dysferlin-mediated repair can be genetically linked (e.g., LGMD2B) [379] or arise during viral myocarditis to exacerbate damage as a cell-cell spread tactic [239]. MG53 is also protective against mechanical damage in cardiomyocytes and this extends to I/R injury where it participates in ischemic preconditioning against subsequent stress [380–382].

### Immune system

The dynamic nature of immune cells poses a challenge for identifying physiological stressors of plasma membrane integrity. However, it appears damage is cell type-specific given the highly specialized functions of immune cells. For instance, the release of neutrophil extracellular traps entails chromatin swelling that provides enough mechanical force to rupture the plasma membrane [383]. Meanwhile, phagocytes can experience damage following interactions with pathogens and unique stressors amidst inflammation (e.g., cholesterol crystals in atherosclerotic plaques) [71, 384]. Immune cell resistance against a particular stressor appears to be highly dependent on plasma membrane composition, as evident by the ability of cytotoxic T lymphocytes to repel perforin-induced damage destined for target cells [385]. Furthermore, certain stressors such as cholesterol-dependent pore-forming toxin display a preference for immune cell lineage (i.e., myeloid versus lymphoid) [35], highlighting the need for such fundamental comparisons when assessing damage.

Contrary to our understanding of how immune cells resolve infection, little is known about how they resolve plasma membrane wounds. In macrophages, lysosomal exocytosis serves a dual purpose: it alleviates membrane damage induced by intracellular pathogens and exports cytotoxic cargo to fend off extracellular bacteria [386, 387]. The membrane remodeling events that accompany repair are not always favorable given the importance of cell surface receptors for immune cell function. In B cells, lysosomal exocytosis can disrupt B cell receptor signaling by promoting the internalization of signaling domains which are required for its activation [185]. Moreover, pore-induced membrane shedding in macrophages can lead to the removal of cytokine receptors from the cell surface resulting in immune suppression [186]. While membrane repair can promote survival in immune cells, it appears to compromise function although how long these defects persist following wound resolution remains unclear.

## Conclusions and perspectives

Plasma membrane integrity is a ubiquitous requirement of cells, regardless of tissue function. Tissue-specific stressors challenge plasma membrane resistance to which cells rely on active repair mechanisms. It is now apparent that cells encounter damage “in sickness and in health” and that membrane damage does not always lead to cell death. Instead, damage is a gradient that can dramatically alter the intracellular landscape and even facilitate intercellular signaling critical to tissue function.

Far more is known about what can damage cells as opposed to how they heal. While membrane repair pathways are generally conserved, it is apparent that cell type-specific adaptations can have broader effects depending on the tissue environment (e.g., mucus secretion in the gut, clotting factor release by endothelium). However, these unique mechanisms, let alone the ability to undergo repair, have yet to be studied in many cell types relevant to disease. Major technical strides towards resolving plasma membrane damage and repair in vivo hold great promise for understanding the importance of this process under physiological stress.

It is now apparent that defects in plasma membrane resistance and repair can cause human disease beyond muscular dystrophies. Genetic mutants may be causative of defects in membrane integrity, with additional genetic factors contributing to disease heterogeneity. Meanwhile in other conditions, environmental stress can be the dominating factor resulting in traumatic or acute injuries. Initial evidence suggests such gene-environment interactions contribute to the pathogenesis of multifactorial diseases which remains a promising avenue of study. Ultimately, understanding plasma membrane damage and repair on a single cell basis can provide therapeutic insight towards wound resolution on a tissue level.

## Data Availability

Not applicable.
